# Prevalence of *Sarcocystis calchasi* in free-ranging host species: *Accipiter* hawks and Common Woodpigeon in Germany

**DOI:** 10.1038/s41598-018-35862-x

**Published:** 2018-12-04

**Authors:** Sylvia L. Parmentier, Kristina Maier-Sam, Klaus Failing, Dirk Enderlein, Achim D. Gruber, Michael Lierz

**Affiliations:** 1 Clinic for Birds, Reptiles, Amphibians and Fish, Justus Liebig University Giessen, Frankfurter Str. 91-93, D-35392 Giessen, Germany; 2 Unit for Biomathematics and Data Processing, Frankfurter Str. 95, D-35392 Giessen, Germany; 3 Institute of Veterinary Pathology, Freie Universität Berlin, Robert-von-Ostertag-Str. 15, D-14163 Berlin, Germany

## Abstract

The apicomplexan parasite *Sarcocystis calchasi* (*S. calchasi*) triggers pigeon protozoal encephalitis, a neurologic disease in columbids. *Accipiter* hawks have been identified as the final host, and Columbidae and Psittaciformes as intermediate hosts. In this study, 368 free-ranging *Accipiter* hawks and 647 free-ranging common woodpigeons were sampled in a country-wide study in order to identify the prevalence of *S. calchasi* in these populations. A semi-nested PCR specific for *S. calchasi* tested positive in 7.3% (4.9–10.5) of submitted samples from *Accipiter* hawks. Juvenile *Accipiter* hawks (13.7%; 7.7–22.0) had a significantly higher infection rate with *S. calchasi* than adult *Accipiter* hawks (5.8%; 2.7–9.3). The prevalence of *S. calchasi* in common woodpigeons was 3.3% (5.4–9.7). Positive pigeons were identified in 14/16 federal states, and a region-dependency was detected, with higher rates of infection in the eastern parts of Germany. The results of this study suggest that the common woodpigeon is a natural reservoir for *S. calchasi*. In a study of one region for four consecutive years, an increase in prevalence was not detected. Findings indicate that the parasite is not newly introduced to Germany, but rather long established. The prevalence suggests that there is a substantial risk of *S. calchasi* infections in other free-ranging as well as captive host species.

## Introduction

The apicomplexan parasite *Sarcocystis calchasi* (*S. calchasi*) is the causative agent of pigeon protozoal encephalitis (PPE)^1^. Like all *Sarcocystis* species, *S. calchasi* has an obligate two-host life cycle^2,3^. While the final host is infected by feeding on tissue containing mature sarcocysts, the intermediate host ingests infectious sporocysts via the faecal-oral route^4^. Outbreaks of PPE were initially reported in racing pigeons (*Columba livia* f. *dom*.) in Berlin, Germany^1^. Subsequently, *S. calchasi* was detected in diseased columbids in the U.S.A. and Japan^5–7^ and psittacines were reported to be further intermediate hosts^8,9^. Recently, *S. calchasi* DNA was detected in the European green woodpecker (*Picus viridus*) and the Great spotted woodpecker (*Dendrocopos major*), but so far, sarcocysts have not been detected in these species^10^. The natural intermediate host reservoir of the parasite has not yet been identified. However, free-ranging columbid species are predicted to be intermediate hosts^3^, but this has not been confirmed to date.

The common woodpigeon (*Columba palumbus palumbus*) is the most common free-ranging pigeon species in Germany, with approximately 9–17 million birds^11^. Common woodpigeon territories are spread in urban and rural areas as well as woodlands throughout the country, with the highest density in North Rhine-Westphalia (NRW)^12,13^. The woodpigeon population is stable to slowly rising. Woodpigeons breeding in Germany show only short migration habits, depending on the season and weather^13,14^. In regions with high amounts of snow during the winter (e.g. black forest), the birds migrate into areas where food resources are available. In regions with a yearlong food supply, the migration distance is often less than 30 kilometres^15–17^. The high density of woodpigeons supports the hypothesis that the population may serve as main natural reservoir of *S. calchasi*^9,18^.

So far, the Northern goshawk (*Accipiter gentilis gentilis*, hereafter goshawk) and the Eurasian sparrowhawk (*Accipiter nisus nisus*, hereafter sparrowhawk) have been identified as final hosts of *S. calchasi*^1,18^. Goshawks are distributed almost completely throughout the Holarctic. Their spread is limited to the north by the forest boundary in the Palaearctic (79° N)^19^ and the southern limit is around 40 °N^20^. In Germany, the number of breeding pairs is estimated to be between 11,500 and 16,500^13^. The population is described as stable with <10% fluctuations^13,21^. Density is low on the coastline, the northeaster part of Germany, sparsely wooded areas in Bavaria and the Alpine foothills. In contrast, the population in NRW and Berlin is high^13,22–24^. Goshawks show a strong migratory behaviour at a juvenile age, whereas adult birds are usually bound to their territories^25^. During the last decades, goshawks have adapted to be a more synanthropic bird, with territories and breeding sites in urban park areas^26^. The goshawk population is considered almost stable, with a small decrease over the past years^13^.

Sparrowhawks are even more common than goshawks, with over 18,000 breeding pairs in Germany^13,27^ and up to 450,000 breeding pairs across Europe^11^. The distribution area of sparrowhawks extends from the Palaearctic to the Mediterranean sub region^28^. The east-west extension reaches from the Canaries to Japan^19^. The sparrowhawk is commonly seen in urban areas and rural lowlands^29,30^. In Germany, a particularly high density is seen in the Münsterland lowland, and a reduced density is documented in the north-east German lowland^13^. Sparrowhawk migration is mostly age- and season-dependent, with reaches into central and southern Europe and North Africa^27,31^.

Both sexes of goshawks prey on adult woodpigeons, mostly during the winter months and while breeding^25,32–35^. Female sparrowhawks prey on birds as large as Eurasian jays (*Garrulus glandarius*) and woodpigeons. Males do not prey on adult woodpigeons, but they are nest predators^36^. The overlapping distributions of goshawks, sparrowhawks and woodpigeons results in a close predator-prey relationship^37–40^.

This close contact between the known final host of *S. calchasi* and the suspected intermediate host reservoir led to the hypothesis that *S. calchasi*’s life cycle is maintained in these species. The present study was designed in order to determine the potential risk to domestic and captive held species, such as racing pigeons and psittacines, as well as to free-ranging intermediate hosts arising from *S. calchasi*-infected free-ranging *Accipiter* hawks and wood pigeons. Additionally, a longitudinal study was designed in order to investigate a potential on-going expansion of *S. calchasi* and to assess if the parasite was either recently introduced into Germany, still spreading or if it was already present with a long-term stable occurrence. In order to investigate the dynamics of a possible current expansion of *S. calchasi*, the woodpigeon was again identified as the host species to be examined, as this species is easily accessible and prevalence increases should be clearly visible.

## Results

### Accipiter hawks

#### Sample collection

In the period of 2012–2016, 368 samples of free-ranging *Accipiter* hawks were collected across Germany. Of these, 119 (32.3%) samples were from goshawks; these consisted of 82 organ and 37 faecal samples. Samples were submitted in every month of the year, but most samples (n = 22) were collected in February. In total, 249 (67.7%) samples originated from sparrowhawks, consisting of 220 organ and 29 faecal samples. Samples were submitted year-round, but most samples (n = 41) were again collected in February.

#### Semi-nested polymerase chain reaction specific to S. calchasi

**Figure 1:**
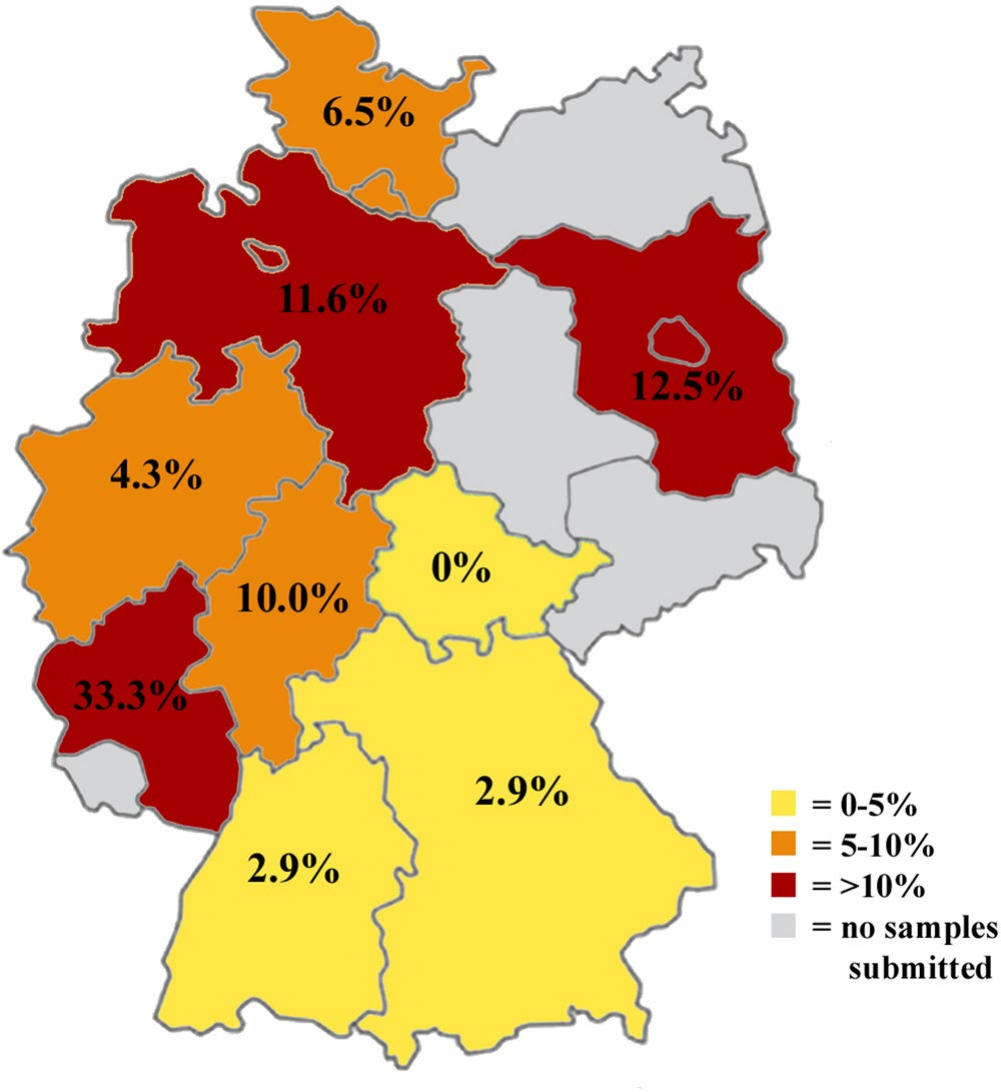
Germany-wide prevalence of *S. calchasi* in *Accipiter* hawks, detected by semi-nested PCR.

*Sarcocystis calchasi* DNA was detected in 27/368 (7.3%; 95% C.I. = 4.9–10.5%) samples from *Accipiter* hawks (Fig. 1).

### Statistical correlations

The age of the *Accipiter* hawks demonstrated a significant dependency on the prevalence of *S. calchasi* (p = 0.022; Odds Ratio = 2.60): Juvenile *Accipiter* hawks were significantly more commonly infected than adult *Accipiter* spp. (Table 1).

**Table 1:** Age-dependency of the *S. calchasi* infection rate in *Accipiter* hawks.

Age	Total	Negative	Positive	Prevalence (%)	95% C.I. (%)	
Juvenile	102	88	14	13.7	7.7	22.0
Adult	208	196	12	5.8	3.0	9.9
No data available	58	57	1			
**Total**	368	341	27	7.3	4.9	10.5

Goshawks were more often infected with *S. calchasi* than sparrowhawks (Odds Ratio = 1.75), although the difference between these species was not statistically significant (p = 0.17) (Table 2). Due to the similar size of female sparrowhawks and male goshawks^41,42^, they share a similar prey spectrum and therefore, were grouped into a single group for the calculation of species-dependency. However, a significant dependency was again not seen (p = 0.65; Odds ratio = 0.71) (Table 3). Neither region, month, quarter or year of submission, type of sample, weight nor sex (within the species) had an effect on *S. calchasi* infection (see Supplementary Table S1). A dependency between the result of the semi-nested PCR and the recorded variables via multi-step logistic regression was not detected. Further steps did not lead to additional significant dependency. Age and species had an independent influence. The entire list of data collected on samples from *Accipiter* hawks can be found as Supplementary Table S2.

**Table 2:** Species-dependency of the *S. calchasi* infection rate in *Accipiter* hawks.

Species	Total	Negative	Positive	Prevalence (%)	95% C.I. (%)	
*A. g. gentilis*	119	107	12	10.1	5.3	17.0
*A. n. nisus*	249	234	15	6.0	3.4	9.7
**Total**	368	341	27	7.3	4.9	10.5

**Table 3:** Dependency of *S. calchasi* infection rate on prey spectrum, with male goshawks and female sparrowhawks grouped together.

	Total	Negative	Positive	Prevalence (%)	95% C.I. (%)	
Female *A. g. gentilis*	49	46	3	6.1	1.3	16.9
Male *A. g. gentilis* + female *A. n. nisus*	186	168	18	9.7	5.8	14.9
Male *A. n. nisus*	71	66	5	7.0	2.3	15.7
No data available	62	61	1			
**Total**	368	341	27	7.3	4.9	10.5

### Common woodpigeons

In the period of 2012–2016, 647 free-ranging woodpigeons were collected and sampled. Forty-five woodpigeons were harvested in the border region between Bavaria and Saxony, therefore, those two federal states were considered as one state in the statistical analysis.

### Prevalence of *S. calchasi*

**Figure 2:**
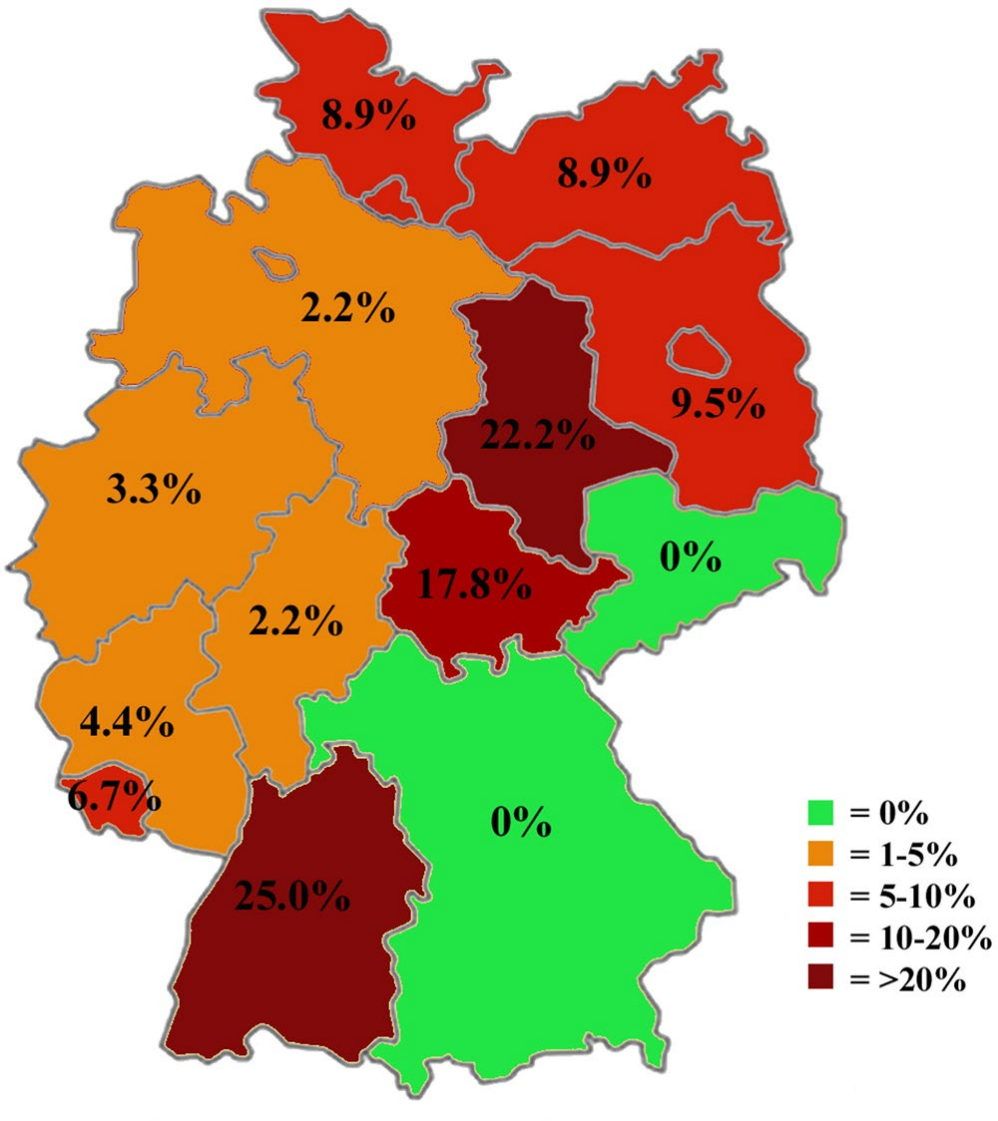
Federal state-based prevalence of *S. calchasi* infection in the common woodpigeon.

DNA of *S. calchasi* was detected in 48 out of 647 (7.4%) sampled woodpigeons. These positive samples originated from 11 federal states. The federal state based prevalence varied from 0% (95% C.I. = 0–6.4%) in Bavaria/Saxony to 25.0% (95% C.I. = 8.7–49.1%) in Baden-Wuerttemberg (Table 4, Fig. 2). There was a highly significant difference between the infection rates of woodpigeons with *S. calchasi* across federal states (p < 0.0001). The weighted average prevalence of *S. calchasi* in Germany within the woodpigeon population was calculated on the basis of hunting bags as weight variables. A hunting bag of a hunting season includes all animals of a species which were hunted within a year and reported to authorities. The hunting authority in each federal state publishes the numbers after every season. The prevalence of *S. calchasi* for Germany is 3.3% (95% C.I. = 5.4–9.7%)(Table 4).

**Table 4:** Sample size, federal state individual record of *S. calchasi* and weighted average prevalence of *S. calchasi* in the common woodpigeon population in Germany.

Federal State	Sample Size	Positive	Negative	Prevalence (%)	95% C.I.	Hunting Bags	Proportion of weight	Weighted average Prevalence
B-W	20	5	15	25.0	8.7–49.1	13,091	0.006	
Bavaria/ Saxony	45	0	45	0	0–6.4	58,527	0.025	
B + Berlin	42	4	38	9.5	2.7–22.6	8,332	0.004	
Hesse	45	1	44	2.2	0.1–11.8	43,836	0.019	
L. Saxony + Bremen	45	1	44	2.2	0.1–11.8	571,507	0.24	
M-W P	45	4	41	8.9	2.5–21.2	2,220	0.0009	
NRW	180	6	174	3.3	1.2–7.1	152,6786	0.65	
Rhineland-Palatine	45	2	43	4.4	0.5–15.1	57,232	0.024	
Saarland	45	3	42	6.7	1.4–18.3	943	0.0004	
Saxony-Anhalt	45	10	35	22.2	11.2–37.1	5,653	0.0024	
S-H + Hamburg	45	4	41	8.9	2.5–21.2	53,829	0.023	
Thuringia	45	8	37	17.8	8.0–32.1	2,976	0.013	
**Total**	647	48	599	7.4	5.4–9.7	2,344,932	1	**3**.**3%**

B-W = Baden-Wuerttemberg; B = Brandenburg; L. Saxony = Lower Saxony; M-W P = Mecklenburg-Western Pomerania; NRW = North Rhine-Westphalia; S-H = Schleswig-Holstein.

### Longitudinal study

From 2012 to 2015, the required number of woodpigeons (n = 45) were collected each year in the same area of NRW. Four-year sampling in NRW revealed no expansion of *S. calchasi* in the woodpigeon population (p = 1.00) (Table 5).

**Table 5:** Detection of *S. calchasi* via semi-nested PCR in woodpigeons collected in North Rhine-Westphalia.

Federal State	Year	Sample Size	Positive
North Rhine-Westphalia	2012	45	2 (4.4%)
	2013	45	1 (2.2%)
	2014	45	1 (2.2%)
	2015	45	2 (4.4%)
**Total**		180	6 (3.3%)

## Discussion

Regionally limited outbreaks of pigeon protozoal encephalitis have been reported in Germany, the United States and Japan^1,5,7^. The evidence presented here strongly suggests a widespread distribution of the parasite in the populations of final and intermediate hosts in Germany, and identifies the woodpigeon as a natural intermediate host reservoir of *S. calchasi*. The woodpigeon population demonstrated a low prevalence of *S. calchasi*. However, the estimated prevalence of certain individual federal states was considerably higher than the weighted average prevalence for Germany. The low average prevalence is mainly influenced by the extraordinary large population size and hunting bag in North Rhine-Westphalia. Therefore, the individual verified prevalence of each federal state should be considered in the risk assessment for each individual region.

The transmission of parasites within their natural host reservoir depends on different relations between predator and prey^43^. Clinical disease increases prey vulnerability significantly, simplifying predator success^40,44^. Prey vulnerability increases after an infection with *S. calchasi*, when neurological symptoms are present. Thus, it seems possible that animals with clinical signs of PPE may be more frequently removed by predators and may be lacking in the samples collected in this study.

Our longitudinal study failed to detect a spread of *S. calchasi* in the woodpigeon population over a four-year period. Thus, it can be assumed that *S. calchasi* had previously established its life cycle within the population. In 2006, *S. calchasi* was detected in racing pigeons in Berlin; the absence of reports prior to this might be a result of a lack of regular histopathological investigations of muscular tissue from birds with central nervous disorders. The chronic phase of PPE is characterised by encephalitis that is usually not associated with protozoan cysts in the brain but in the skeletal muscles^4^. As skeletal muscles may not be examined routinely in cases of neurologic disease, the presence of *S. calchasi* may have been undetected in previous cases of PPE in Germany.

*Sarcocystis calchasi* was detected by PCR in 7.3% of all samples from *Accipiter* hawks. This contrasts with the findings of a previous study: in a small *Accipiter* hawk population located in the Berlin area, 31 of 50 (62.0%) goshawks and 14 of 20 (71.4%) sparrowhawks were positive for *S. calchasi* DNA according to PCR^18^. The higher infection rates noted in the literature may indicate local and/or temporal variances or may be a result of small sample sizes. Fluctuations in the incidence of parasitic diseases are commonly seen^45,46^. The Berlin area may be an endemic hotspot for *S. calchasi* since its prevalence in the woodpigeon population is well above the average of the surrounding federal states (Brandenburg, Mecklenburg-Western Pomerania, Saxony-Anhalt) (Fig. 2).

This study confirms the previously reported^18^ occurrence of *S. calchasi* in both species of *Accipiter* hawks across Germany. Both *Accipiter* hawks show a distinct sexual dimorphism in body size^47,48^, resulting in different prey preferences^33,49^. While both female and male goshawks prey on pigeons, male sparrowhawks do not often prey on columbids, due to their small body size^41,50–53^. As a result, a higher prevalence of *S. calchasi* had been suspected in goshawks and in female sparrowhawks, compared to male sparrowhawks. Indeed, a higher infection rate was detected in goshawks (Table 2), but sex of the *Accipiter* hawks did not significantly influence prevalence of *S. calchasi*, similarly to a previous study^18^. Re-grouping into three groups of the same body size with similar prey preferences did not have an impact on prevalence either (Table 3). Therefore, intermediate host species other than columbids can be assumed. Even though most *Sarcocystis* spp. are described as having a narrow host spectrum, some species profit from either multiple intermediate or final hosts (e.g. *S. neurona*, *S. falcatula* and *S. riley*)^54–63^. Besides columbids, several psittacine species have already been confirmed as intermediate hosts of *S. calchasi*^8,9^ and recently, DNA of *S. calchasi* was detected in two Picidae species in Germany^10^. As a result, other avian prey species, such as small songbirds (Passeri) or woodpeckers (Picidae), may serve as additional reservoirs for *S. calchasi*.

DNA of *S. calchasi* was detected in 13.7% of the juvenile and 5.8% of the adult *Accipiter* hawks submitted. The higher rate of infection in juvenile birds may be explained by their less developed immune system, making them less resistant to parasitic infections^64–67^. In addition, juvenile *Accipiter* hawks are less effective hunters than older birds of the same species^32,68–70^; they may feed more frequently on carrion, seriously ill or dying prey. Therefore, they may more frequently consume intermediate hosts infected with *S. calchasi* or hosts infected with higher doses of *S. calchasi*.

This large-scale cross-sectional study demonstrates that *Sarcocystis calchasi* is prevalent throughout Germany in the final host population, as well as in the free-ranging intermediate host. It can be assumed that the common woodpigeon serves as a natural intermediate host reservoir for the parasite. Therefore, there is a risk of outbreaks of *S. calchasi*-induced PPE in domestic pigeons and captive psittacines across Germany.

## Methods

### *Accipiter* hawks – Sample collection and statistical calculation

#### Sample collection

Within a period of four years (2012–2016), 368 samples from *Accipiter* hawks were submitted by rescue centres, veterinary clinics, taxidermists, forestry admissions and veterinary diagnostic laboratories. Samples consisted of whole carcasses, intestine samples from wildlife casualties, and faecal samples (see Supplementary Table S2); the latter were collected during the first three days of captivity prior to any antiparasitic treatment. Portions of 200 mg faecal samples were stored for DNA extraction. Carcasses and organ samples were submitted fresh, cooled or frozen. Fresh and cooled samples were investigated directly. Frozen submissions were defrosted at room temperature. A necropsy of the carcasses was performed and tissue samples were taken from two locations of the small intestine and one location of the large intestine and either quick-frozen or immediately submitted for DNA extraction. Sex, age, weight, month and year of collection were recorded in a questionnaire. The questionnaire was completed either during necropsy or, in the case of submission of faecal samples and tissue, by the dispatcher.

#### Statistical calculation

The sample size for *Accipiter* hawks was calculated to gain data for an individual-based prevalence. Calculation was based on the prevalence detected in Berlin, Germany (60%)^18^ and conducted with BiAS for Windows (Version 9.05–02/2010)^71^. To acquire a 95% confidence interval (C.I.) with an accepted deviation of ± 5%, a sample size of 368 samples was calculated. A sampling plan based on region was not necessary due to the large distribution range of *Accipiter* and the high migration rate of the species^13^. All samples collected over the length of the study were included. Statistical data analysis was based on a multiple stepwise logistic regression, with the following variables: ‘species’, ‘location’, ‘type of sample’, ‘age’, ‘sex’, ‘prey spectrum’, ‘weight’, ‘month of collection’, ‘calendar quarter of collection’ and ‘year of collection’. The analysis was carried out with the statistical program package BMDP (BMDP Statistical Software, Inc.^72^).

### Common woodpigeon – Sample collection and statistical calculation

#### Sample collection

In Hesse and North-Rhine Westphalia, samples were taken at a hunting spot, while woodpigeons from Rhineland-Palatine and Lower Saxony were submitted cooled and sampled at the Clinic for Birds, Reptiles, Amphibians and Fish, Justus Liebig University Giessen, Germany (hereafter clinic). Woodpigeons collected from Mecklenburg-Vorpommern, Schleswig-Holstein, Bavaria, Baden-Württemberg, Saxony-Anhalt, Thuringia and Saarland were submitted frozen to the clinic. They were defrosted at room temperature prior to having samples taken. Biopsies of 2 × 2 × 2 cm were taken from the muscular tissue of the upper and lower breast muscle and were either quick-frozen or immediately submitted for DNA extraction.

#### Statistical calculation

The prevalence of *S. calchasi* in woodpigeons is presently unknown. Initial studies failed to detect the parasite in this species^3^. Therefore, the prevalence was predicted to be rather low (5%). To identify the prevalence of *S. calchasi* in the woodpigeon population, a cross sectional study was designed. The population of woodpigeons is not evenly spread in Germany, but varies between the federal states^13^. The spatial distribution of *S. calchasi*, therefore, can only be estimated via stratified sampling. In the present study, stratification was done by federal states. All federal states were included in the study, even if the hunting bag in some federal states is very low (Table 4). City-states were attributed to the surrounding states. The hunting bags served as a basis for calculating the minimum sample size per federal state, because of the correlation between bag size and the regional population of free-ranging pigeons. The requested specific confidence interval probability was defined as 95%, with a tolerable deviation of $\pm$ 2%. Using BiAS for Windows (Version 9.05–02/2010)^71^, a sample size of 455 samples was calculated. As the estimated prevalence is considered inaccurate, and an even lower true prevalence cannot be ruled out, the sample size was raised to n ≥500. As a premise, we defined the probability of detection of at least one positive bird in each federal state to be at least 0.9, resulting in the formula: P(X $\ge 1$ | Prevalence = 5%) ≥0.90. Cannon and Roe’s charts identified a necessary sample size of 45 samples in each federal state^73^. If the collection was not feasible due to a small harvest in the period from 2012 until spring 2015, woodpigeon populations were sampled during the winter hunting season of 2015 within the migration distance of 25 kilometres at the borders of any federal state which had yet acquired enough samples. With 13 federal states in total, this led to a required sample size of 585 samples (13 × 45).

In order to assess the on-going expansion of *S. calchasi*, a longitudinal study was conducted in North Rhine-Westphalia, which harbours the largest woodpigeon population^13^ and hunting bags in Germany. Sample collection of woodpigeons was extended to at least three consecutive years in NRW. The sample size was aligned to the total sample size per federal state of 45 samples per year. Comparisons of the prevalence between the sampled years was done using Fisher’s exact test^74^.

In the subsequent evaluation, extrapolation for a nationwide prevalence was based on the hunting bags for the period of sample collection in each federal state. Comparisons of the prevalence between federal states was done using Fisher’s exact test^74^. Additionally, the 95% C.I. of the prevalence and the odds ratio (OR) were calculated.

### Semi-nested polymerase chain reaction specific to *Sarcocystis calchasi*

DNA was extracted (DNeasy Blood & Tissue Kit; Qiagen, Hilden, Germany) from intestinal samples of the *Accipiter* hawks and muscular tissues of the woodpigeons. Faecal samples were treated according to the protocol for the Stool Extraction Kit (Qiagen, Hilden, Germany). Afterwards, DNA concentration was measured (260–280 nm) (NanoDrop 2000c Spectrophotometer; Thermo Fisher Scientific, Wilmington, DE, USA) and, if necessary, diluted to < 5 ng/μl according to manufacturer instructions. A semi-nested PCR was performed according to a previously described protocol^75^. *Sarcocystis calchasi* DNA extracted from the Berlin strain^18^ was used in a 10-fold serial dilution as a positive control, and DNAse-free water was included as a non-template control.

### Statement of ethical approval

The samples of the current study were obtained from animals that had died or were killed for reasons that are not related with the purpose of this study. Samples were collected from wood pigeons which had been killed for hunting purposes. Carcasses and organ samples from *Accipiter* hawks were provided by clinics, rescue centres or other institutions, where these animals had died or had been humanely killed due to their fatal clinical condition. Faecal samples from living *Accipiter* hawks were collected during regular cage cleaning and did not involve any further handling of these birds. Therefore, this study was not subject to ethics review.

## Electronic supplementary material

Dataset 1

## Data Availability

The datasets generated during and/or analysed during the current study are available from the corresponding author on reasonable request.
