# Supplementary material for: Prevalence of Sarcocystis calchasi in free-ranging host species: Accipiter hawks and Common Woodpigeon in Germany
Source: Sci Rep. 2018 Dec 4;8:17610. doi: 10.1038/s41598-018-35862-x (PMC6279811; doi:10.1038/s41598-018-35862-x)
Supplement: Supplementary file 1 — Dataset 1 [file 41598_2018_35862_MOESM1_ESM.pdf]

Supplementary Information on

Prevalence of *Sarcocystis calchasi* in free-ranging host species: *Accipiter* hawks and Common Woodpigeon in Germany

Sylvia L. Parmentier, Kristina Maier-Sam, Klaus Failing, Dirk Enderlein, Achim D. Gruber, Michael Lierz

Supplementary Table S1: p-values and raw odds ratios of variables recorded from *Accipiter* hawks without a significant impact on a *S. calchasi* infection

Variables	p-Value	Odds Ratio
Species	0.17	1.75
Sex	0.27	1.58
Region	0.32	0.91
Sample type	0.35	0.55
Quarter of submission	0.46	1.47
Prey Spectrum	0.65	0.71
Month of submission	0.83	0.64
Weight	0.85	1.00
Year of submission	1.00	1.00

Supplementary Table S2: Recorded data on samples from *Accipiter* hawks

Within a period of four years 368 *Accipiter* hawks were collected, data were recorded via a questionnaire and a semi-nested PCR specific for *S. calchasi* tested positive 27of the submitted samples

Consecutive Number	PCR	Species	Federal State	Region	Type of Sample	Age	Sex	Prey Spectrum	Weight	Month of Submission	Year of Submission
GV 001	neg	G	L. Saxony	North German Plain	o	juv	♀	3	594	Feb	2016
GV 002	neg	G	Hesse	Central Upland and Scarplands	o	juv	♂	2	500	Jul	2012
GV 003	neg	S	Hesse	Central Upland and Scarplands	o	juv	♀	2	178	Aug	2012
GV 004	neg	S	Hesse	Central Upland and Scarplands	o	juv	♂	1	164	Aug	2012
GV 005	neg	S	R-P	Central Upland and Scarplands	o	ad	♂	1	175	Nov	2012
GV 006	neg	S	Hesse	Central Upland and Scarplands	o	ad	♂	1	131	Dec	2012
GV 007	neg	G	L. Saxony	North German Plain	o	ad	♀	3	915	Nov	2012
GV 008	neg	G	NRW	North German Plain	f	ad	♀	3	*	Oct	2012
GV 009	neg	G	NRW	North German Plain	f	ad	♂	2	*	Oct	2012
GV 010	neg	G	NRW	North German Plain	f	ad	♀	3	*	Oct	2012
GV 011	neg	S	NRW	North German Plain	f	ad	♀	2	*	Nov	2012
GV 012	neg	S	NRW	North German Plain	f	juv	♀	2	230	Oct	2012
GV 013	neg	S	Bavaria	Alpine Forland	o	juv	♂	1	128	Nov	2012
GV 014	neg	S	L. Saxony	North German Plain	o	ad	♀	2	170	Dec	2012
GV 015	neg	S	L. Saxony	North German Plain	o	juv	♀	2	195	Dec	2012
GV 016	neg	S	L. Saxony	North German Plain	o	ad	♂	1	134	Dec	2012
GV 017	neg	S	Hesse	Central Upland and Scarplands	o	ad	♀	2	188	Dec	2012
GV 019	neg	S	Bavaria	Alpine Forland	o	ad	♀	2	160	Dec	2012
GV 020	neg	G	Hesse	Central Upland and Scarplands	o	juv	♂	2	*	Aug	2012
GV 021	neg	G	Hesse	Central Upland and Scarplands	o	juv	♂	2	*	Oct	2012

Consecutive Number	PCR	Species	Federal State	Region	Type of Sample	Age	Sex	Prey Spectrum	Weight	Month of Submission	Year of Submission
GV 022	neg	S	Hesse	Central Upland and Scarplands	o	ad	♂	1	211	Mar	2012
GV 023	neg	G	Hesse	Central Upland and Scarplands	o	juv	♀	3	772	Apr	2012
GV 024	neg	S	Hesse	Central Upland and Scarplands	o	juv	♂	2	198	Nov	2013
GV 025	neg	S	Hesse	Central Upland and Scarplands	o	juv	♂	1	125	Dec	2013
GV 026	neg	S	Hesse	Central Upland and Scarplands	o	juv	♂	1	204	Apr	2013
GV 027	neg	S	Hesse	Central Upland and Scarplands	o	juv	♀	2	112	Jan	2013
GV 028	neg	S	Hesse	Central Upland and Scarplands	o	ad	♂	1	110	Jan	2013
GV 029	neg	S	Hesse	Central Upland and Scarplands	o	juv	♀	2	*	Apr	2013
GV 030	neg	S	Hesse	Central Upland and Scarplands	o	juv	♀	2	*	Feb	2013
GV 031	neg	S	Hesse	Central Upland and Scarplands	o	juv	♀	2	*	Oct	2013
GV 032	neg	S	Bavaria	Alpine Forland	o	ad	♀	2	166	Mar	2013
GV 033	neg	S	Hesse	Central Upland and Scarplands	o	juv	♀	2	198	*	2013
GV 034	neg	S	Hesse	Central Upland and Scarplands	o	juv	♂	2	241	*	2013
GV 035	neg	S	Hesse	Central Upland and Scarplands	o	juv	♀	2	204	Apr	2013
GV 036	neg	S	Hesse	Central Upland and Scarplands	o	ad	♀	2	*	Sep	2013
GV 037	neg	S	Hesse	Central Upland and Scarplands	o	juv	♂	1	*	Oct	2013
GV 038	neg	G	Hesse	Central Upland and Scarplands	o	ad	♀	3	*	Sep	2013
GV 039	**pos**	G	Hesse	Central Upland and Scarplands	o	juv	♀	3	1000	Jan	2013
GV 040	neg	S	L. Saxony	North German Plain	o	ad	♀	2	242	Feb	2013
GV 041	neg	G	Bavaria	Alpine Forland	f	ad	*	0	660	Feb	2013
GV 042	neg	G	NRW	North German Plain	f	juv	♂	2	650	Feb	2013
GV 043	neg	S	B-W	Alpine Forland	o	juv	♂	1	142	Feb	2013
GV 044	neg	G	Hesse	Central Upland and Scarplands	o	ad	♂	2	760	Feb	2013
GV 045	neg	S	Bavaria	Alpine Forland	o	ad	♀	2	162	Feb	2013

Consecutive Number	PCR	Species	Federal State	Region	Type of Sample	Age	Sex	Prey Spectrum	Weight	Month of Submission	Year of Submission
GV 046	neg	S	L. Saxony	North German Plain	o	ad	♂	1	126	Mar	2013
GV 047	neg	S	L. Saxony	North German Plain	o	*	♀	2	99	Mar	2013
GV 048	neg	S	L. Saxony	North German Plain	o	ad	♀	2	210	Mar	2013
GV 049	neg	S	L. Saxony	North German Plain	o	ad	♀	2	151	Mar	2013
GV 050	neg	G	L. Saxony	North German Plain	o	ad	♀	3	520	Mar	2013
GV 051	neg	G	Hesse	Central Upland and Scarplands	o	ad	♂	2	*	Feb	2013
GV 052	neg	G	Hesse	Central Upland and Scarplands	o	ad	♂	2	*	Jan	2013
GV 053	neg	S	Hesse	Central Upland and Scarplands	o	ad	♂	1	*	Oct	2013
GV 054	neg	G	Hesse	Central Upland and Scarplands	o	ad	♂	2	*	Jun	2013
GV 055	**pos**	G	Hesse	Central Upland and Scarplands	o	juv	♂	2	*	Feb	2013
GV 056	neg	S	Hesse	Central Upland and Scarplands	o	ad	♀	2	*	Oct	2013
GV 057	neg	G	Hesse	Central Upland and Scarplands	o	juv	♂	2	*	Mar	2013
GV 058	neg	S	L. Saxony	North German Plain	f	ad	♀	2	*	Mar	2013
GV 059	neg	S	L. Saxony	North German Plain	o	ad	♂	1	114	Mar	2013
GV 060	neg	S	L. Saxony	North German Plain	f	ad	♀	2	*	Mar	2013
GV 061	**pos**	S	NRW	North German Plain	o	juv	♂	1	120	Apr	2013
GV 062	neg	S	Bavaria	Alpine Forland	o	ad	♂	1	116	Apr	2013
GV 063	neg	S	NRW	North German Plain	f	juv	♂	1	150	Apr	2013
GV 064	neg	S	NRW	North German Plain	o	ad	♂	1	109	Apr	2016
GV 065	neg	S	Bavaria	Alpine Forland	o	ad	♂	1	107	May	2013
GV 067	neg	S	Hesse	Central Upland and Scarplands	o	ad	♀	2	110	May	2013
GV 068	neg	G	Hesse	Central Upland and Scarplands	o	ad	♀	3	630	Jul	2013
GV 069	neg	G	Hesse	Central Upland and Scarplands	o	ad	♂	2	1250	Jul	2013
GV 070	neg	S	Hesse	Central Upland and Scarplands	o	ad	♀	2	234	Jun	2013

Consecutive Number	PCR	Species	Federal State	Region	Type of Sample	Age	Sex	Prey Spectrum	Weight	Month of Submission	Year of Submission
GV 071	neg	G	Hesse	Central Upland and Scarplands	o	ad	♂	2	*	Feb	2013
GV 072	neg	S	Hesse	Central Upland and Scarplands	o	juv	♀	2	*	Jan	2013
GV 073	neg	S	L. Saxony	North German Plain	o	ad	♀	2	255	Jul	2013
GV 074	neg	S	Hesse	Central Upland and Scarplands	o	ad	♀	2	191	Aug	2013
GV 075	neg	S	Hesse	Central Upland and Scarplands	o	ad	♂	1	126	Aug	2013
GV 076	neg	S	B-W	Alpine Forland	o	juv	♀	2	151	Aug	2013
GV 077	neg	G	Bavaria	Alpine Forland	o	juv	♀	3	452	Aug	2013
GV 078	neg	S	B-W	Alpine Forland	o	juv	♀	2	151	Aug	2013
GV 079	neg	S	Berlin	North German Plain	o	juv	♂	2	230	Sep	2013
GV 080	neg	S	Berlin	North German Plain	o	juv	♂	1	152	Sep	2013
GV 081	**pos**	S	Berlin	North German Plain	o	ad	♀	2	217	Sep	2013
GV 082	neg	S	Berlin	North German Plain	o	ad	♂	1	187	Sep	2013
GV 083	neg	S	Berlin	North German Plain	o	ad	♀	2	252	Sep	2013
GV 084	neg	S	Berlin	North German Plain	o	ad	♀	2	255	Sep	2013
GV 085	neg	S	Berlin	North German Plain	o	ad	♀	2	187	Sep	2013
GV 086	neg	S	Hesse	Central Upland and Scarplands	f	*	*	0	*	Sep	2013
GV 087	neg	G	Hesse	Central Upland and Scarplands	f	ad	♀	3	1114	Sep	2013
GV 088	neg	S	B-W	Alpine Forland	o	juv	♂	1	170	Dec	2013
GV 089	neg	S	Hesse	Central Upland and Scarplands	o	ad	♀	2	94	Dec	2013
GV 090	neg	S	Hesse	Central Upland and Scarplands	o	ad	♀	2	210	Dec	2013
GV 091	neg	G	Hesse	Central Upland and Scarplands	f	ad	♀	3	*	Dec	2013
GV 092	neg	S	Thuringia	Central Upland and Scarplands	o	ad	♂	1	115	Dec	2013
GV 093	neg	S	B-W	Alpine Forland	o	ad	♀	2	199	Jan	2014
GV 094	neg	S	B-W	Alpine Forland	o	ad	♀	2	230	Dec	2014

Consecutive Number	PCR	Species	Federal State	Region	Type of Sample	Age	Sex	Prey Spectrum	Weight	Month of Submission	Year of Submission
GV 095	**pos**	S	B-W	Alpine Forland	o	juv	♂	1	85	Jan	2014
GV 096	neg	S	B-W	Alpine Forland	o	ad	♀	2	277	Jan	2014
GV 097	neg	G	Hesse	Central Upland and Scarplands	f	ad	♀	3	772	Jan	2014
GV 098	**pos**	S	Hesse	Central Upland and Scarplands	o	ad	♀	2	*	Feb	2014
GV 099	**pos**	G	Bavaria	Alpine Forland	o	ad	♂	2	*	Mar	2014
GV 100	neg	S	Hesse	Central Upland and Scarplands	o	juv	♂	1	*	Nov	2014
GV 101	neg	S	Hesse	Central Upland and Scarplands	o	ad	♀	2	240	Apr	2014
GV 102	neg	S	Hesse	Central Upland and Scarplands	o	juv	♀	2	132	Aug	2014
GV 103	neg	S	Hesse	Central Upland and Scarplands	o	juv	♂	1	*	Aug	2014
GV 104	neg	S	Hesse	Central Upland and Scarplands	o	ad	♂	1	*	Feb	2014
GV 105	neg	S	Hesse	Central Upland and Scarplands	o	juv	♀	2	*	Dec	2014
GV 106	**pos**	S	Hesse	Central Upland and Scarplands	o	juv	♂	1	*	Jan	2014
GV 107	neg	S	Hesse	Central Upland and Scarplands	o	juv	♀	2	*	Jan	2014
GV 108	**pos**	G	Hesse	Central Upland and Scarplands	o	ad	♂	2	618	Oct	2014
GV 109	**pos**	G	Hesse	Central Upland and Scarplands	o	juv	♂	2	463	Oct	2014
GV 110	neg	G	Hesse	Central Upland and Scarplands	o	juv	♂	2	*	Nov	2014
GV 111	**pos**	G	Hesse	Central Upland and Scarplands	o	juv	♀	3	*	Oct	2014
GV 112	**pos**	S	Hesse	Central Upland and Scarplands	o	ad	♂	1	*	Feb	2014
GV 113	**pos**	S	Hesse	Central Upland and Scarplands	o	juv	♂	1	120	Oct	2014
GV 114	neg	S	Hesse	Central Upland and Scarplands	o	ad	♀	2	*	Jan	2014
GV 115	neg	S	Hesse	Central Upland and Scarplands	o	juv	♀	2	*	Jul	2014
GV 116	neg	S	Hesse	Central Upland and Scarplands	o	juv	♀	2	*	Mar	2014
GV 117	neg	S	Hesse	Central Upland and Scarplands	o	juv	♀	2	*	Feb	2014
GV 118	neg	S	Hesse	Central Upland and Scarplands	o	ad	♂	2	209	Mar	2014

Consecutive Number	PCR	Species	Federal State	Region	Type of Sample	Age	Sex	Prey Spectrum	Weight	Month of Submission	Year of Submission
GV 119	neg	S	Hesse	Central Upland and Scarplands	o	ad	♂	1	141	Apr	2014
GV 120	neg	S	Hesse	Central Upland and Scarplands	o	ad	♂	1	110	Apr	2014
GV 121	neg	S	B-W	Alpine Forland	o	ad	♀	2	223	May	2014
GV 122	neg	S	B-W	Alpine Forland	o	ad	♂	1	95	May	2014
GV 123	neg	S	B-W	Alpine Forland	o	ad	♂	1	135	May	2014
GV 124	neg	S	B-W	Alpine Forland	o	ad	♀	2	222	May	2014
GV 125	neg	S	NRW	North German Plain	o	ad	♀	2	210	Jul	2014
GV 126	neg	S	NRW	North German Plain	o	ad	♀	2	225	Feb	2016
GV 127	neg	S	NRW	North German Plain	o	ad	♂	1	94	Feb	2016
GV 128	neg	S	NRW	North German Plain	o	ad	♀	2	158	Feb	2016
GV 129	neg	S	NRW	North German Plain	o	ad	♂	1	124	Jul	2014
GV 130	neg	G	Hamburg	Mecklenburg Coastal Lowland	f	juv	♂	2	*	Jul	2014
GV 131	neg	G	Hamburg	Mecklenburg Coastal Lowland	f	ad	♂	2	*	Jul	2014
GV 132	neg	S	Hamburg	Mecklenburg Coastal Lowland	f	ad	♀	2	*	Jul	2014
GV 133	**pos**	G	Hamburg	Mecklenburg Coastal Lowland	f	juv	♂	2	*	Jul	2014
GV 134	neg	G	Hamburg	Mecklenburg Coastal Lowland	f	juv	♀	3	*	Jul	2014
GV 135	neg	G	NRW	North German Plain	o	juv	♀	3	*	Jun	2014
GV 136	neg	G	Hamburg	Mecklenburg Coastal Lowland	f	juv	♂	2	*	Jul	2014
GV 137	neg	S	S-H	Mecklenburg Coastal Lowland	f	juv	♀	2	*	Jul	2014
GV 138	neg	G	Hamburg	Mecklenburg Coastal Lowland	f	juv	♀	3	*	Jul	2014
GV 139	neg	S	NRW	North German Plain	o	ad	♂	1	139	Aug	2014
GV 140	neg	S	Hesse	Central Upland and Scarplands	o	ad	♀	2	347	Aug	2014
GV 141	neg	S	Hesse	Central Upland and Scarplands	o	ad	♀	2	*	Aug	2014
GV 142	neg	S	B-W	Alpine Forland	f	ad	♀	2	*	Aug	2014

Consecutive Number	PCR	Species	Federal State	Region	Type of Sample	Age	Sex	Prey Spectrum	Weight	Month of Submission	Year of Submission
GV 143	neg	G	B-W	Alpine Forland	f	juv	♂	2	*	Aug	2014
GV 144	neg	S	B-W	Alpine Forland	f	juv	♂	1	*	Aug	2014
GV 145	neg	G	L. Saxony	North German Plain	o	ad	♂	2	600	Aug	2014
GV 146	**pos**	S	L. Saxony	North German Plain	o	ad	♀	2	200	Jun	2014
GV 147	neg	G	L. Saxony	North German Plain	o	ad	♂	2	525	May	2014
GV 148	**pos**	S	NRW	North German Plain	f	juv	♀	2	220	Sep	2014
GV 149	neg	G	Hamburg	Mecklenburg Coastal Lowland	f	juv	♂	2	700	Aug	2014
GV 150	neg	G	NRW	North German Plain	f	ad	♀	3	700	Aug	2014
GV 151	neg	S	Hesse	Central Upland and Scarplands	f	juv	♀	2	230	Aug	2014
GV 152	neg	G	NRW	North German Plain	o	juv	♀	3	900	Aug	2014
GV 153	neg	S	L. Saxony	North German Plain	f	ad	♀	2	263	Feb	2014
GV 154	neg	S	B-W	Alpine Forland	o	ad	♂	1	*	Oct	2014
GV 155	neg	S	Bavaria	Alpine Forland	o	*	*	0	*	Jul	2014
GV 156	neg	S	Bavaria	Alpine Forland	o	*	*	0	*	Aug	2014
GV 157	neg	S	Bavaria	Alpine Forland	o	*	*	0	*	Aug	2014
GV 158	neg	S	Bavaria	Alpine Forland	o	*	*	0	*	Aug	2014
GV 159	neg	G	Bavaria	Alpine Forland	o	*	*	0	*	Aug	2014
GV 160	neg	S	Hesse	Central Upland and Scarplands	o	ad	♂	2	223	Oct	2014
GV 161	neg	S	NRW	North German Plain	o	ad	♀	2	226	Oct	2014
GV 162	neg	S	Hesse	Central Upland and Scarplands	o	juv	♀	2	95	Oct	2014
GV 163	neg	G	Hesse	Central Upland and Scarplands	o	ad	♀	3	510	Oct	2014
GV 164	neg	G	Hesse	Central Upland and Scarplands	o	ad	♀	3	846	Oct	2014
GV 165	neg	S	Hesse	Central Upland and Scarplands	o	ad	♂	1	116	Oct	2014
GV 166	neg	G	NRW	North German Plain	o	ad	*	0	*	Nov	2014

Consecutive Number	PCR	Species	Federal State	Region	Type of Sample	Age	Sex	Prey Spectrum	Weight	Month of Submission	Year of Submission
GV 167	neg	G	Hesse	Central Upland and Scarplands	f	ad	*	0	*	Nov	2014
GV 168	neg	G	Bavaria	Alpine Forland	o	ad	♀	3	686	Oct	2014
GV 169	neg	G	Bavaria	Alpine Forland	o	ad	♂	2	633	Oct	2014
GV 170	neg	S	Bavaria	Alpine Forland	o	ad	♂	1	92	Sep	2014
GV 171	neg	S	Bavaria	Alpine Forland	o	ad	♂	1	173	Sep	2014
GV 172	neg	S	Bavaria	Alpine Forland	o	ad	♀	2	181	Oct	2014
GV 173	neg	S	Bavaria	Alpine Forland	o	ad	♀	2	98	Oct	2014
GV 174	neg	S	Bavaria	Alpine Forland	o	ad	♂	2	223	Oct	2014
GV 175	neg	G	Hamburg	Mecklenburg Coastal Lowland	f	ad	♀	3	1050	Dec	2015
GV 176	neg	G	Hamburg	Mecklenburg Coastal Lowland	f	ad	♂	2	724	Dec	2015
GV 177	neg	S	NRW	North German Plain	o	*	*	0	*	*	2015
GV 178	neg	S	NRW	North German Plain	o	*	*	0	*	*	2015
GV 179	neg	S	NRW	North German Plain	o	*	*	0	*	*	2015
Gv 180	neg	S	NRW	North German Plain	o	*	*	0	*	*	2015
Gv 181	neg	S	NRW	North German Plain	o	*	*	0	*	*	2015
Gv 182	neg	S	NRW	North German Plain	o	*	*	0	*	*	2015
Gv 183	neg	S	NRW	North German Plain	o	*	*	0	*	*	2015
GV 184	neg	S	NRW	North German Plain	o	*	*	0	*	*	2015
GV 185	neg	S	NRW	North German Plain	o	*	*	0	*	*	2015
GV 186	neg	S	NRW	North German Plain	o	*	*	0	*	*	2015
GV 187	neg	S	NRW	North German Plain	o	*	*	0	*	*	2015
GV 188	neg	S	NRW	North German Plain	o	*	*	0	*	*	2015
GV 189	neg	S	NRW	North German Plain	o	*	*	0	*	*	2015
GV 190	neg	S	NRW	North German Plain	o	*	*	0	*	*	2015

Consecutive Number	PCR	Species	Federal State	Region	Type of Sample	Age	Sex	Prey Spectrum	Weight	Month of Submission	Year of Submission
GV 191	neg	S	NRW	North German Plain	o	*	*	0	*	*	2015
GV 192	neg	S	NRW	North German Plain	o	*	*	0	*	*	2015
GV 193	neg	S	NRW	North German Plain	o	*	*	0	*	*	2015
GV 194	neg	S	NRW	North German Plain	o	*	*	0	*	*	2015
GV 195	neg	S	NRW	North German Plain	o	*	*	0	*	*	2015
GV 196	neg	G	NRW	North German Plain	o	*	*	0	*	*	2015
GV 197	neg	S	NRW	North German Plain	o	*	*	0	*	*	2015
GV 198	neg	S	NRW	North German Plain	o	*	*	0	*	*	2015
GV 199	neg	G	NRW	North German Plain	o	*	*	0	*	*	2015
GV 200	neg	S	NRW	North German Plain	o	*	*	0	*	*	2015
GV 201	neg	S	NRW	North German Plain	o	*	*	0	*	*	2015
GV 202	neg	S	NRW	North German Plain	o	*	*	0	*	*	2015
GV 203	neg	S	NRW	North German Plain	o	*	*	0	*	*	2015
GV 204	neg	G	NRW	North German Plain	o	*	*	0	*	*	2015
GV 205	neg	G	NRW	North German Plain	o	ad	*	0	*	*	2015
GV 206	neg	G	NRW	North German Plain	o	*	*	0	*	*	2015
GV 207	**pos**	G	NRW	North German Plain	o	*	*	0	*	*	2015
GV 208	neg	G	NRW	North German Plain	o	*	*	0	*	*	2015
GV 209	neg	G	NRW	North German Plain	o	*	*	0	*	*	2015
GV 210	neg	G	NRW	North German Plain	o	*	*	0	*	*	2015
GV 211	neg	G	Bavaria	Alpine Forland	o	juv	♀	3	600	Jan	2015
GV 212	neg	G	Hesse	Central Upland and Scarplands	o	*	*	0	992	Jan	2015
GV 213	neg	G	Hesse	Central Upland and Scarplands	o	*	*	0	*	Jan	2015
GV 214	neg	S	Hesse	Central Upland and Scarplands	o	*	*	0	*	Jan	2015

Consecutive Number	PCR	Species	Federal State	Region	Type of Sample	Age	Sex	Prey Spectrum	Weight	Month of Submission	Year of Submission
GV 215	neg	G	Hesse	Central Upland and Scarplands	o	*	*	0	*	Oct	2015
GV 216	neg	S	Bavaria	Alpine Forland	o	*	*	0	*	Jan	2015
GV 217	neg	S	Bavaria	Alpine Forland	o	*	*	0	*	Jan	2015
GV 218	neg	S	Bavaria	Alpine Forland	o	*	*	0	*	Jan	2015
GV 219	neg	S	Bavaria	Alpine Forland	o	*	*	0	*	Jan	2015
GV 220	neg	S	Bavaria	Alpine Forland	o	*	*	0	*	Jan	2015
GV 221	neg	S	Bavaria	Alpine Forland	o	*	*	0	*	*	2015
GV 222	neg	S	NRW	North German Plain	o	*	*	0	*	*	2015
GV 223	neg	S	NRW	North German Plain	o	*	*	0	*	*	2015
GV 224	neg	G	Bavaria	Alpine Forland	o	*	*	0	*	*	2015
GV 225	neg	G	NRW	North German Plain	o	*	*	0	*	*	2015
GV 226	neg	S	Hesse	Central Upland and Scarplands	o	*	*	0	*	*	2015
GV 227	neg	S	Hesse	Central Upland and Scarplands	o	*	*	0	*	*	2015
GV 228	neg	S	L. Saxony	North German Plain	o	juv	♂	1	*	*	2015
GV 229	neg	S	Hesse	Central Upland and Scarplands	f	*	*	0	100	Feb	2015
GV 230	neg	G	Hesse	Central Upland and Scarplands	f	*	*	0	*	Feb	2015
GV 231	neg	S	L. Saxony	North German Plain	f	ad	♀	2	250	Feb	2015
GV 232	neg	G	NRW	North German Plain	f	ad	♂	2	*	Feb	2015
GV 233	neg	G	Hamburg	Mecklenburg Coastal Lowland	o	ad	♂	2	580	Feb	2015
GV 234	neg	G	Hamburg	Mecklenburg Coastal Lowland	o	ad	♀	3	380	Feb	2015
GV 235	neg	G	Hamburg	Mecklenburg Coastal Lowland	o	ad	♀	3	570	Feb	2015
GV 236	neg	G	Hamburg	Mecklenburg Coastal Lowland	o	ad	♂	1	640	Feb	2015
GV 237	neg	G	Hamburg	Mecklenburg Coastal Lowland	o	ad	♀	3	1320	Oct	2015
GV 238	neg	S	Hamburg	Mecklenburg Coastal Lowland	o	ad	♀	2	194	Feb	2015

Consecutive Number	PCR	Species	Federal State	Region	Type of Sample	Age	Sex	Prey Spectrum	Weight	Month of Submission	Year of Submission
GV 239	neg	S	Hamburg	Mecklenburg Coastal Lowland	o	ad	♂	2	195	Feb	2015
GV 240	neg	S	Hamburg	Mecklenburg Coastal Lowland	o	ad	♂	2	250	Feb	2015
GV 241	neg	S	Hamburg	Mecklenburg Coastal Lowland	o	ad	♀	2	130	Feb	2015
GV 242	neg	S	Hamburg	Mecklenburg Coastal Lowland	o	ad	♀	2	270	Feb	2015
GV 243	neg	S	Hamburg	Mecklenburg Coastal Lowland	o	ad	♀	2	300	Feb	2015
GV 244	neg	S	Hamburg	Mecklenburg Coastal Lowland	o	ad	♂	1	77	Feb	2015
GV 245	neg	S	Hamburg	Mecklenburg Coastal Lowland	o	ad	♂	1	139	Mar	2015
GV 246	neg	S	Hamburg	Mecklenburg Coastal Lowland	o	ad	♀	2	191	Dec	2015
GV 247	neg	S	Hesse	Central Upland and Scarplands	o	ad	♀	2	*	Jan	2015
GV 248	neg	G	NRW	North German Plain	f	juv	♂	2	660	Feb	2015
GV 249	neg	G	L. Saxony	North German Plain	o	ad	♂	2	480	Feb	2015
GV 250	neg	S	L. Saxony	North German Plain	o	ad	♀	2	208	Feb	2015
GV 251	neg	S	L. Saxony	North German Plain	o	ad	♀	2	237	Feb	2015
GV 252	neg	S	L. Saxony	North German Plain	o	ad	♀	2	165	Feb	2015
GV 253	neg	S	L. Saxony	North German Plain	o	ad	♂	1	103	Feb	2015
GV 254	neg	S	L. Saxony	North German Plain	o	ad	♀	2	149	Feb	2015
GV 255	neg	S	L. Saxony	North German Plain	o	ad	♂	1	98	Feb	2015
GV 256	neg	S	L. Saxony	North German Plain	o	ad	♀	2	119	Feb	2015
GV 257	neg	S	L. Saxony	North German Plain	o	ad	♂	1	120	Feb	2015
GV 258	neg	G	Hesse	Central Upland and Scarplands	o	ad	♂	2	624	Feb	2015
GV 259	**pos**	S	L. Saxony	North German Plain	o	ad	♀	2	*	Feb	2015
GV 260	neg	G	Hesse	Central Upland and Scarplands	f	ad	♂	2	812	Mar	2015
GV 261	neg	G	B-W	Alpine Forland	o	ad	♀	3	*	Mar	2015
GV 262	neg	S	L. Saxony	North German Plain	o	juv	♀	2	162	Nov	2014

Consecutive Number	PCR	Species	Federal State	Region	Type of Sample	Age	Sex	Prey Spectrum	Weight	Month of Submission	Year of Submission
GV 263	neg	S	L. Saxony	North German Plain	o	ad	♀	2	250	Nov	2014
GV 264	neg	S	L. Saxony	North German Plain	o	ad	♀	2	218	Sep	2015
GV 265	neg	S	L. Saxony	North German Plain	o	ad	♀	2	230	Jan	2015
GV 266	neg	S	B-W	Alpine Forland	o	ad	♂	1	113	Apr	2015
GV 267	neg	G	Hesse	Central Upland and Scarplands	o	ad	♀	3	690	Jun	2015
GV 268	neg	S	Hesse	Central Upland and Scarplands	o	ad	♂	1	121	Oct	2015
GV 269	neg	S	NRW	North German Plain	f	juv	♀	2	260	Jul	2015
GV 270	neg	S	B-W	Alpine Forland	o	juv	♀	2	240	Jul	2015
GV 271	neg	S	B-W	Alpine Forland	o	ad	♀	2	230	Jul	2015
GV 272	neg	G	Hesse	Central Upland and Scarplands	o	juv	♀	3	790	Jan	2015
GV 273	neg	G	NRW	North German Plain	f	juv	♀	3	930	Jun	2015
GV 274	neg	S	NRW	North German Plain	f	juv	♀	2	250	Feb	2015
GV 275	neg	S	L. Saxony	North German Plain	f	ad	♀	2	*	Jul	2015
GV 276	neg	G	Hesse	Central Upland and Scarplands	f	ad	♀	3	*	Jul	2015
GV 277	**pos**	G	Hesse	Central Upland and Scarplands	o	juv	♂	2	*	Feb	2015
GV 278	neg	G	Hesse	Central Upland and Scarplands	o	juv	♀	3	*	Dec	2014
GV 279	neg	G	Hesse	Central Upland and Scarplands	f	ad	♂	2	524	Jul	2015
GV 280	neg	G	B-W	Alpine Forland	o	juv	♀	3	600	Jul	2015
GV 281	neg	G	NRW	North German Plain	f	juv	♂	2	466	Jul	2015
GV 282	neg	G	NRW	North German Plain	f	juv	♂	2	495	Jul	2015
GV 283	**pos**	G	Hesse	Central Upland and Scarplands	f	ad	♀	3	*	Aug	2015
GV 284	**pos**	G	Hamburg	Mecklenburg Coastal Lowland	o	juv	♀	2	550	Aug	2015
GV 285	neg	S	Hamburg	Mecklenburg Coastal Lowland	o	juv	♂	1	140	Aug	2015
GV 286	neg	S	Hamburg	Mecklenburg Coastal Lowland	o	ad	♀	2	203	Aug	2015

Consecutive Number	PCR	Species	Federal State	Region	Type of Sample	Age	Sex	Prey Spectrum	Weight	Month of Submission	Year of Submission
GV 287	neg	S	Hamburg	Mecklenburg Coastal Lowland	o	juv	♂	1	141	Aug	2015
GV 288	neg	S	Hamburg	Mecklenburg Coastal Lowland	o	ad	♂	1	145	Aug	2015
GV 289	neg	S	Hesse	Central Upland and Scarplands	f	juv	♀	2	230	Aug	2015
GV 290	neg	S	NRW	North German Plain	o	juv	♂	1	130	Aug	2015
GV 291	neg	S	NRW	North German Plain	o	juv	♀	2	226	Aug	2015
GV 292	neg	S	NRW	North German Plain	o	ad	♀	2	160	Sep	2015
GV 293	neg	S	B-W	Alpine Forland	o	juv	♀	2	165	Sep	2015
GV 294	neg	S	B-W	Alpine Forland	o	ad	♂	1	139	Aug	2015
GV 295	neg	S	B-W	Alpine Forland	o	juv	♀	2	146	Sep	2015
GV 296	neg	G	Berlin	Central Upland and Scarplands	o	ad	♀	3	860	Aug	2015
GV 298	neg	G	B-W	Alpine Forland	o	juv	♂	2	565	Aug	2015
GV 299	neg	G	NRW	North German Plain	f	juv	♂	2	614	Aug	2015
GV 300	neg	G	Hesse	Central Upland and Scarplands	o	juv	♂	2	517	Dec	2015
GV 301	neg	G	NRW	North German Plain	o	ad	♀	3	1080	Dec	2015
GV 302	neg	G	Hesse	Central Upland and Scarplands	o	ad	♀	3	1240	Dec	2015
GV 303	neg	S	B-W	Alpine Forland	o	juv	♂	2	185	Dec	2015
GV 304	neg	S	B-W	Alpine Forland	o	ad	♂	1	135	Dec	2015
GV 305	neg	S	NRW	North German Plain	f	ad	♂	1	*	Dec	2015
GV 306	neg	G	L. Saxony	North German Plain	f	ad	♀	3	*	Dec	2015
GV 307	neg	S	B-W	Alpine Forland	o	ad	♀	2	381	Dec	2015
GV 308	neg	S	B-W	Alpine Forland	o	juv	♀	2	210	Sep	2015
GV 309	neg	G	Hesse	Central Upland and Scarplands	o	ad	♂	2	560	Dec	2014
GV 310	neg	S	Hesse	Central Upland and Scarplands	o	juv	♂	1	156	Oct	2015
GV 311	neg	G	B-W	Alpine Forland	o	juv	♀	3	480	Jul	2015

Consecutive Number	PCR	Species	Federal State	Region	Type of Sample	Age	Sex	Prey Spectrum	Weight	Month of Submission	Year of Submission
GV 312	neg	S	NRW	North German Plain	o	juv	♂	1	150	Oct	2015
GV 313	neg	S	NRW	North German Plain	o	juv	♂	1	135	Oct	2015
GV 314	neg	S	Hesse	Central Upland and Scarplands	o	ad	♀	2	255	Sep	2015
GV 315	neg	S	Bavaria	Alpine Forland	f	ad	♀	2	209	Oct	2015
GV 316	neg	G	Hesse	Central Upland and Scarplands	o	ad	♂	2	840	Oct	2015
GV 317	neg	G	B-W	Alpine Forland	o	ad	♂	2	*	Oct	2015
GV 318	neg	G	Hesse	Central Upland and Scarplands	o	juv	♀	3	900	Oct	2015
GV 319	neg	S	NRW	North German Plain	o	ad	♀	2	294	Oct	2015
GV 320	neg	G	NRW	North German Plain	o	ad	♀	3	*	Oct	2015
GV 321	neg	G	Hesse	Central Upland and Scarplands	o	ad	♀	3	*	Oct	2015
GV 322	neg	S	NRW	North German Plain	o	ad	♀	2	278	Nov	2015
GV 323	neg	S	NRW	North German Plain	o	ad	♀	2	250	Nov	2015
GV 324	neg	S	NRW	North German Plain	o	ad	♂	1	124	Nov	2015
GV 325	neg	S	NRW	North German Plain	o	ad	♀	2	181	Nov	2015
GV 326	neg	S	NRW	North German Plain	o	ad	♂	1	130	Nov	2015
GV 327	neg	S	NRW	North German Plain	o	ad	♀	2	251	Nov	2015
GV 328	neg	S	NRW	North German Plain	o	ad	♀	2	292	Nov	2015
GV 329	neg	S	NRW	North German Plain	o	ad	♂	1	134	Nov	2015
GV 330	neg	S	NRW	North German Plain	o	ad	♀	2	177	Nov	2015
GV 331	neg	S	NRW	North German Plain	o	ad	♀	2	184	Nov	2015
GV 332	neg	S	NRW	North German Plain	o	ad	♂	1	102	Nov	2015
GV 333	neg	S	NRW	North German Plain	o	ad	♀	2	225	Nov	2015
GV 334	**pos**	G	Hesse	Central Upland and Scarplands	o	juv	♂	2	780	Dec	2015
GV 335	neg	S	Hesse	Central Upland and Scarplands	o	juv	♀	2	148	Nov	2015

Consecutive Number	PCR	Species	Federal State	Region	Type of Sample	Age	Sex	Prey Spectrum	Weight	Month of Submission	Year of Submission
GV 336	neg	S	Hesse	Central Upland and Scarplands	o	ad	♀	2	247	Nov	2015
GV 337	neg	G	Hesse	Central Upland and Scarplands	o	juv	♀	3	*	Nov	2015
GV 338	neg	S	Hesse	Central Upland and Scarplands	o	ad	♀	2	193	Dec	2015
GV 339	neg	G	Hesse	Central Upland and Scarplands	o	ad	♂	3	935	Dec	2015
GV 340	neg	S	B-W	Alpine Forland	o	ad	♂	1	111	Jan	2016
GV 341	neg	G	B-W	Alpine Forland	o	ad	♂	2	546	Jan	2016
GV 342	neg	S	B-W	Alpine Forland	o	ad	♀	2	169	Dec	2016
GV 343	neg	G	R-P	Central Upland and Scarplands	o	ad	♀	3	905	Dec	2016
GV 344	neg	G	Hesse	Central Upland and Scarplands	f	ad	♀	3	964	Jan	2015
GV 345	neg	S	Hesse	Central Upland and Scarplands	o	ad	♂	1	127	Jan	2016
GV 346	neg	G	Hesse	Central Upland and Scarplands	o	juv	♂	2	603	Jan	2016
GV 347	neg	S	NRW	North German Plain	o	ad	♀	2	208	Jan	2016
GV 348	neg	S	NRW	North German Plain	f	juv	♀	2	210	Jan	2016
GV 349	neg	G	Hamburg	Mecklenburg Coastal Lowland	o	ad	♂	3	956	Feb	2016
GV 350	**pos**	S	R-P	Central Upland and Scarplands	o	ad	♀	2	190	Feb	2016
GV 351	neg	S	NRW	North German Plain	f	ad	♀	2	194	Feb	2016
GV 352	neg	S	B-W	Alpine Forland	f	ad	♂	1	107	Feb	2016
GV 353	neg	S	L. Saxony	North German Plain	f	ad	♀	2	253	Oct	2016
GV 354	neg	S	L. Saxony	North German Plain	f	ad	♀	2	191	Jul	2016
GV 355	neg	S	Hesse	Central Upland and Scarplands	f	ad	♀	2	*	Feb	2016
GV 356	neg	G	Bavaria	Alpine Forland	f	ad	♂	2	500	Feb	2016
GV 357	neg	S	Hesse	Central Upland and Scarplands	f	ad	♀	2	269	Feb	2016
GV 358	neg	G	L. Saxony	North German Plain	f	ad	♂	2	*	Feb	2016
GV 359	neg	S	Hesse	Central Upland and Scarplands	o	ad	♂	1	*	Feb	2016

Consecutive Number	PCR	Species	Federal State	Region	Type of Sample	Age	Sex	Prey Spectrum	Weight	Month of Submission	Year of Submission
GV 360	neg	S	Hesse	Central Upland and Scarplands	o	ad	♀	2	*	Feb	2016
GV 361	neg	G	Hesse	Central Upland and Scarplands	f	ad	♀	3	*	Feb	2016
GV 362	**pos**	S	L. Saxony	North German Plain	o	ad	♀	2	270	Feb	2016
GV 364	neg	G	Hesse	Central Upland and Scarplands	f	ad	*	0	*	Feb	2016
GV 365	**pos**	S	NRW	North German Plain	o	juv	♀	2	194	Oct	2016
GV 366	neg	S	NRW	North German Plain	f	ad	♀	2	184	Oct	2016
GV 367	**pos**	S	L. Saxony	North German Plain	o	ad	♂	2	220	Feb	2016
GV 368	**pos**	S	L. Saxony	North German Plain	o	ad	♂	2	187	Feb	2016
GV 369	neg	S	Bavaria	Alpine Forland	o	ad	♀	2	201	Mar	2016
GV 370	neg	S	Bavaria	Alpine Forland	o	ad	♀	2	176	Mar	2016
GV 371	neg	S	Bavaria	Alpine Forland	o	ad	♂	1	96	Mar	2016
GV 372	neg	S	Bavaria	Alpine Forland	o	juv	♂	2	193	Mar	2016

Pos, positive; neg, negative; G, Northern Goshawk; S, Eurasian Sparrowhawk; L. Saxonym, Lower Saxony; R-P, Rhineland-Palatine; NRW, North Rhine-Westphalia; B-W, Baden-Wuerttemberg;S-H, Schleswig-Holstein; o, organ; f, faecal; juv, juvenile; ad, adult; 1, male sparrowhawks; 2, female sparrowhawks + male goshawks; 3, female goshawks; * no data available
